# M. Powell Lawton Award Presentation

**DOI:** 10.1093/geroni/igaa057.3195

**Published:** 2020-12-16

**Authors:** Debra Dobbs

## Abstract

“The lecture will be given by the2019 recipient, Barbara Resnick, PhD, CRNP, FGSA of the University of Maryland. The session will also include the presentation of the2020 Lawton Award to recipient Sara J. Czaja, PhD, FGSA. The M. Powell Lawton Award is presented annually to an individual who has made outstanding contributions from applied research that has benefited older people and their care. The Lawton Award is generously funded by the Polisher Research Institute of the Madlyn and Leonard Abramson Center for Jewish Life.”

